# Therapeutic potential of peptides from Ole e 1 in olive-pollen allergy

**DOI:** 10.1038/s41598-019-52286-3

**Published:** 2019-11-04

**Authors:** David Calzada, Lucía Cremades-Jimeno, María Ángeles de Pedro, Selene Baos, Manuel Rial, Joaquín Sastre, Joaquín Quiralte, Fernando Florido, Carlos Lahoz, Blanca Cárdaba

**Affiliations:** 1 Immunology Department, IIS-Fundación Jiménez Díaz, UAM, Madrid, Spain; 2 Allergy Department, Fundación Jiménez Díaz, Madrid, Spain; 3 CIBERES, CIBER of Respiratory Diseases, Madrid, Spain; 4 Allergy Department, Vírgen del Rocío University Hospital, Seville, Spain; 5 Allergy Department, San Cecilio University Hospital, Granada, Spain

**Keywords:** Experimental models of disease, Molecular medicine

## Abstract

Olive-pollen allergy is one of the leading causes of respiratory allergy in Mediterranean countries and some areas of North America. Currently, allergen-specific immunotherapy is the only etiophatogenic treatment. However, this approach is not fully optimal, safe, or effective. Thus, efforts continue in the search for novel immunotherapy strategies, being one of the most promising the use of peptides derived from major allergens. This work tries to determine the therapeutic potential and safety of 5 dodecapeptides derived from the main allergen of olive-pollen allergy, Ole e 1. The immunomodulatory capacity of these peptides was studied using peripheral blood mononuclear cells (PBMCs) obtained from 19 olive-pollen-allergic patients and 10 healthy controls. We determined the capacity of these peptides to inhibit the proliferative response toward olive-pollen allergenic extract and to induce the regulatory cytokines, IL-10 and IL-35. To test the safety and absence of allergenicity of the peptides, the basophil activation was analyzed by flow-cytometry, using peripheral blood. The results showed that two of five peptides inhibited near to 30% the proliferative response against the total olive-pollen allergenic extract in olive-pollen-allergic patients. Inhibition increased to nearly 35% when the 5 peptides were used in combination. In both cases, a statistically significant induction of IL-10 and IL-35 secretion was observed in the supernatants of allergic patients PBMCs cultures. None of the 5 peptides induced basophil activation and cross-link inflammatory cell-bound IgE. In conclusion, these results open up new possibilities in the treatment of olive-pollen allergy, which could solve some of the problems facing current therapy approaches.

## Introduction

Peripheral T-cell tolerance of environmental substances is tightly controlled by the immune system due to the functional deactivation caused by specific T-cell subtypes, generically called regulatory T-cells (Tregs), which have an immunosuppressive effect. The secretion of traditional regulatory cytokines, such as interleukin (IL)-10, and novel related molecules, such as IL-35, are some of the most important mechanisms involved in the immune response^1^. However, under certain environmental conditions, an imbalance in these regulatory mechanisms can induce allergic diseases, promoting allergen-specific IgE production as well as activation and recruitment of pro-inflammatory cells in target organs^1,2^.

Allergic diseases are a global health problem, affecting up to 25% of the population in industrialized societies^3,4^. To date, allergen-specific immunotherapy (AIT) is the only treatment that can change the course of these diseases. Although AIT has been used for over 100 years^5^, treatment with whole-allergen extracts is not without its drawbacks, as AIT can cause local and systemic adverse events and may produce new IgE sensitization to other allergens present in the extract. Furthermore, the lengthy treatment duration (3–5 years), frequent administration, and high treatment cost are among the other disadvantages of AIT^6,7^. For these reasons, there is a need for safer and more effective AIT strategies.

Immunotherapy based on modified allergens or peptides has been considered since more than 20 years, however, the last advances in molecular biology of allergens and the mechanisms implicated in their recognition and the modulation of their responses have increased the number of experimental and clinical trials to try to improve the immunotherapy guidelines, and the peptides therapy is one of the most promising treatments in course^8^. This therapy for allergic disease involves the use of soluble allergen fragments of variable lengths and peptide-based vaccines, which could solve several of the main problems facing conventional AIT. This approach also offers additional advantages, such as high stability, ease of purification, standardization, and low production costs. In addition, the therapeutic dose may be used without the need of dose escalation^6,9^. Peptide vaccines are designed based on the primary structure of the allergen. There are two types of peptides, depending on the length of the fragments and their capacity to induce tolerance: IgE-mediated peptides, which are long peptides, having a length of 20 to 40 amino acids (aa); and synthetic peptide immuno-regulatory epitopes (SPIRE), consisting of T-cell tolerizing peptides made up of smaller peptide units (10 to 17 aa)^10,11^.

One of the most prevalent types of pollinosis in Mediterranean countries and some areas of North America is caused by the pollen of olive trees. This pollen mainly induces nasal and conjunctival symptoms and may cause asthma exacerbation in areas with high antigenic load, such as Andalusia, a region in Spain where olive tree is widely cultivated and with particularly high pollen counts during the pollen season (5,000 grains/m^3^, with peaks reaching more than 10,000 grains/m^3^). The incidence of pollinosis is high during pollen season, lasting from mid-April to the end of June^12^. The main allergen, Ole e 1, recognized by almost 80% of allergic subjects, is a 145-aa glycoprotein with microheterogeneity of sequence, highly dependent on the olive cultivar analyzed^13,14^ but a high degree of sequence homology and IgE cross-reactivity to the main allergens in other *Oleaceae*-family pollens, such as lilac, ash, and privet^15^. Ole e 1 has at least 4 B-cell epitopes^16^ and 2 regions, aa 91 to 102 and aa 119 to 130, which were defined as immunodominant T-cell epitopes, or the regions mainly recognized by olive-pollen-allergic patients, able to induce a T cell-proliferative response with no IgE-binding capability^17^. In addition, a pilot study of our group showed how stimulation with Ole e 1 peptides derived from the sequence of Ole e 1 obtained after Edman degradation^18^, induced a different cytokine profile in peripheral blood mononuclear cells (PBMCs) from olive-pollen-allergic patients compared to nonallergic subjects. Two peptides that included the aa11-33 Ole e 1 region were mainly recognized by nonallergic subjects and induced IL-10 levels *in vitro*, which is why they were postulated as possible immunoregulatory peptides^19^. More recently, we explored how these Ole e 1-derived peptides affect gene-expression profile and we examined their *in vitro* capacity to modulate the Th1/Th2 response. In our previous article, we reported that these peptides were capable of modulating some genes implicated in the tolerance response, which could be of interest in the effort to develop a new immunomodulatory treatment^20^.

In this report, we expand the body of research into the use of short Ole e 1-derived peptides as a new promising method in the treatment of olive-pollen allergy. We carried out an *in vitro* analysis of the ability of combinations of Ole e 1 immunomodulatory peptides to prevent or reverse the olive pollen response and their safety (absence of basophil activation). We also analyzed the implication of the classical regulatory cytokine, IL-10, as well as the new regulatory cytokine, IL-35, in this modulation, to establish the potential of these peptides as future immunotherapeutic tools for this disorder.

IL-35, the newest member of the IL-12 family, is secreted mainly by stimulated Tregs^21^. It is a heterodimer composed of IL12 p35 and EBI3^22^, but, in contrast to the rest of IL-12 family (IL-12, IL-23, IL-27) that are involved in the pro-inflammatory response, IL-35 mediates immunological functions by suppressing inflammatory immune response. Besides, this cytokine was analyzed in this study because EBI3 was one of the genes that we previously find as specifically modulated by peptides 2 and 3 and, considered as a possible therapeutic target for olive-pollen allergy^20^. Our results point that Ole e 1 peptides could induce a regulatory response mediated by IL-35 and IL-10, being able to reduce the olive pollen response, and reinforcing the idea of these peptides as useful therapeutic tools for preventing these respiratory disorders.

## Material and Methods

### Subjects

The study population comprised 19 untreated olive-pollen-allergic patients, including 13 asthmatic olive-pollen-allergic subjects and 6 nonasthmatic subjects with the same allergy. Ten nonallergic subjects were used as healthy controls. All patients were diagnosed and recruited from the allergy departments of two hospitals located in Seville and Granada, both in Andalusia, a region of southern Spain chosen for its high olive-pollen counts. Nonallergic control subjects were healthy and had no history of respiratory allergy. Biological samples from subjects were obtained outside the pollen season, from October to December, when environmental pollen counts are low.

Olive-pollen-allergic patients fulfilled the following criteria established in accordance with EAACI recommendations: rhinitis or rhinitis with asthma from April to June, with a positive skin prick test for *O. europaea* pollen extract (ALK Abelló, Madrid, Spain) and no previous immunotherapy (EAACI, 1989). The exclusion criteria were as follows: age under 16 years, less than 10 years’ residence in the study area, and corticosteroid or anti-histaminic treatment.

### Total and specific IgE antibody measurements

Ten to 20 ml of peripheral heparin blood samples and 10 ml of blood without anticoagulant were obtained from each study subject for cellular and serological analysis. Total serum IgE levels were determined using an IgE enzyme immunoassay (Phadia, Uppsala, Sweeden), *O. europaea* pollen-specific IgE and Ole e 1-specific IgE antibody levels were quantified by UNI-CAP system (Phadia). Levels of specific IgE > 0.35 kUA/l were considered positive.

### Olive-tree pollen extract and Ole e 1 peptide purification

Olive-tree pollen was obtained from Allergom AB, Sweden. The pollen (5% w/v) was extracted with 50 mM ammonium bicarbonate, pH 8.0, containing 1 mM phenyl-methyl-sulfonyl-fluoride, followed by centrifugation at 12 000 × g for 20 min at 4 °C. The lyophilized supernatant was stored at −20 °C.

Ole e 1 dodecapeptides were synthesized (purity > 90%) according to the Ole e 1 amino-acid sequence^18^. The peptides used were as follows: peptide (P) 2 (aa11-22: FHIQGQVYCDTC), P3 (aa22-33: CRAGFITELSEF), P10 (aa91-102: NEIPTEGWAKPS), P12 (aa109-120: TVNGTTRTVNPL), and P13 (aa119-130: PLGFFKKEALPK), all as previously were described^17,19,20^.

### PBMC isolation and culture

PBMCs were isolated from heparin-containing peripheral blood samples by gradient centrifugation on Lymphoprep (Comercial Rafer, Zaragoza, Spain) following the manufacturer’s instructions. PBMCs were cultured in triplicate in a P96-U-bottom plate (Costar, New York, USA) at a density of 1 × 10^6^ cells/ml, in RPMI 1640 (Lonza, Verviers, Belgium) supplemented with 10% fetal bovine serum (FBS) inactivated (Lonza), 1% glutamine (Gibco, Carlsbad, California, USA),1% peni-streptomycin (Gibco), and 1 mM sodium pyruvate (Gibco) in the absence or presence of olive-pollen stimuli, over 6 days, 37 °C, 5% CO_2_ in humidified air, as was previously described^17,19,20^.

**Figure 1:**
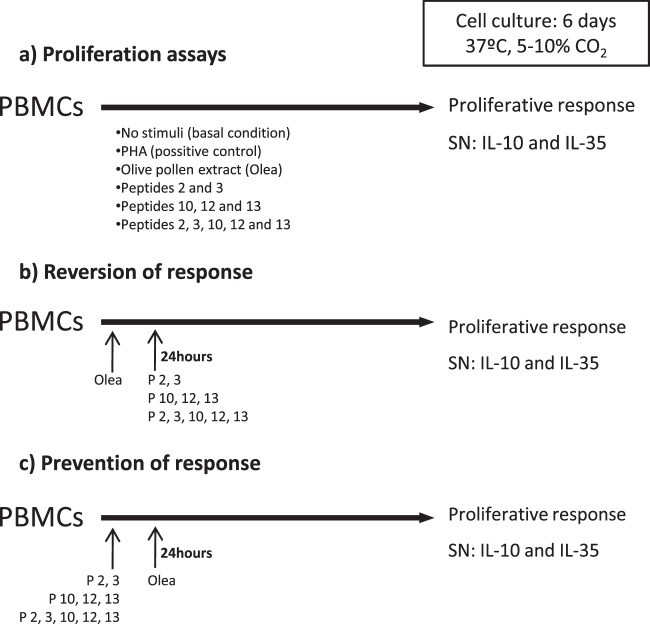
Scheme of proliferation and/or inhibition of PBMCs assays. PBMCs from the study subjects were cultured with different combinations of peptides or/and olive-pollen extract (Olea) as indicate the figure, for 6 days, in order to determine: the proliferative response (**a**), reversion of the response (**b**) or inhibition response (**c**). Three different combinations of peptides were used: peptides 2 and 3 (immunoregulatory region); peptides 10, 12, and 13 (immunodominant region), and both groups of peptides. After 6 days of stimulation, proliferative response was measured. Also, supernatants (SN) were collected for a subsequent study of cytokine secretion.

The different stimuli were as follows: olive-pollen extract (*Olea europaea*, 25 µg/ml) named “Olea”, peptides 2 and 3 (P 2 + 3, 5 µg/ml), peptides 10, 12, and 13 (P 10 + 12 + 13, 5 µg/ml), and the combination of all of peptides, peptides 2, 3 (5 µg/ml) and peptides 10, 12, and 13 (5 µg/ml). PHA (10 µg/ml) was used as a positive control. Concentration and combination of stimuli were based on previous assays^17^. All stimuli were reconstituted in RPMI medium. Figure 1a presents an overview of the protocol used.

After stimulation and plate centrifugation (10 min, 720 g), supernatants were used to measure soluble cytokine levels and cells were then resuspended in 100 µl of fresh culture medium. To quantify the levels of proliferation, 20 µl of Cell Titer 96 Aqueous One Solution Cell Proliferation Assay (Promega, Madison, USA) was added to each well, following the manufacturer’s instructions. After 90 minutes, absorbance at 490 nm was measured with the microplate reader *Infinite F200* (Tecan, Männedorf, Switzerland), and the average absorbance values from three wells per experimental condition were calculated. The stimulation index was determined as the ratio of average absorbance of each condition and basal condition (without stimulation). Only those assays that showed a positive value for the ratio of PHA/basal were considered valid. Also, it was measured the cell viability by Trypan blue (Gibco) method, being always higher than 80%.

### Inhibition of PBMC proliferative response

In order to demonstrate the immunomodulation properties of the peptides, we designed two types of inhibition proliferation assay. An overview of the protocol used is given in Fig. 1b,c. Optimal inhibition conditions were established by time curve studies (see Supplementary Fig. S1).

Reversion of response assays: PBMCs (1 × 10^6^ cells/ml) were cultured in the presence of olive-pollen extract for 6 days; after 24 hours, peptides 2 and 3 (P2 + 3, 5 µg/ml), peptides 10, 12, and 13 (P10, 12, and 13, 5 µg/ml), or the combination of all peptides (peptides 2, 3 (5 µg/ml) and peptides 10, 12, and 13 (5 µg/ml)), as appropriate, were added to the medium of these PBMCs.

Prevention of response assays: PBMCs (1 × 10^6^ cells/ml) were cultured in the presence of peptides 2 and 3 (P2 + 3, 5 µg/ml), peptides 10, 12, and 13 (P10, 12, and 13, 5 µg/ml), or the combination of all peptides (peptides 2, 3 (5 µg/ml) and peptides 10, 12, and 13 (5 µg/ml)), for six days, adding 25 µg/ml of olive-pollen extract to the medium after 24 hours.

In both cases, absorbance was measured and compared to stimulation with olive pollen extract only, that was named “Olea” condition, whose proliferation was taken to be 100%.

### Measurement of soluble cytokine levels

All the supernatants collected in the cellular assays were stored at −80 °C until use to measure soluble cytokine levels. Levels of two soluble regulatory cytokines—IL-10 and IL-35—were analyzed. IL-10 was measured in supernatant of proliferation and inhibition-of-response assays of all patients and controls, using ELISA (ImmunoTools, Germany), although in two patients the levels of IL-10 were under the detection threshold (9.8 pg/ml). IL-35 was measured in the supernatants of 9 control assays and 17 olive-pollen-allergic patient assays, also by ELISA (Elabscience, Houston, Texas, USA), but one patient was desestimated because the levels of IL-35 were over the detection threshold (1000 pg/ml). IL-4 (ImmunoTools, Germany), IFN-γ (ImmunoTools, Germany) and TGF-β (R&D Systems, Minneapolis, USA) were also measured in a percentage of the population studied (6 healthy control subjects and 7 allergic subjects), but without any conclusive result related with the stimuli (data not shown).

### Basophil activation test

As fresh heparinized blood is needed for this test, 3 olive-pollen-allergic patients and 3 healthy control subjects were recruited outside pollen season from the *Fundación Jiménez Díaz* hospital in Madrid, Spain, with the same criteria than the population of study. Informed consent was obtained from each subject, and ethical approval was granted by the ethical and research committee of the hospital. Total IgE and *O. europaea*- and Ole e 1-specific IgE-antibody determinations were performed in the allergy department of the hospital.

Basophil activation test was performed to test peptide safety using the BasoFlowEx® Kit (EXBIO Diagnostics, Huissen, Netherlands) according to the manufacturer’s instructions. Briefly, 100 µl of heparinized whole blood samples was incubated for 15 minutes at 37 °C with different stimuli: control (anti-IgE monoclonal antibody as positive control sample), 3 concentrations of olive-pollen extract (5, 25, and 125 µg/ml), 3 concentrations of peptides 2 and 3 and/or 10, 12, and 13 (1, 5, and 25 µg/ml), or nothing (negative control). The concentrations tested were based in the optimal extract or peptide dose conditions, 25 µg/ml for olive pollen extract and 5 µg/ml for peptides respectively, as was previously described [16], checking two more conditions: 5 times higher and 5 times lower. Samples were then labeled with PE-anti human CD203c antibody to select the basophil population, and FITC-anti human CD63 antibody to identify the activated basophils. Samples were immediately analyzed by Facs Canto II flow cytometer (Becton-Dickinson BD, San Jose, California, USA).

Data acquisition was carried out on 100,000 events, and the results of basophil activation were analyzed with the Infinicyt™flow cytometry software (Cytognos, Salamanca, Spain). Only those assays with a positive control higher than 20% were considered reliable. Activation > 15% by stimuli was considered positive, according to the manufacturer’s instructions.

### Statistical analysis

Statistical analyses were carried out using GraphPad InStat software. IgE levels and the results of proliferation and ELISA assays were analyzed by Mann-Whitney test. Prevention and reversion of response assays were analyzed by Wilconxon test, comparing their medians to the olive-pollen condition median (100%).

### Ethical approval

Written informed consent in accordance with the Declaration of Helsinki was obtained from each subject. Ethical approval for the study was obtained from the ethical and research committees of the participating hospitals (IIS-Fundación Jiménez Díaz, *Vírgen del Rocío and San Cecilio* University Hospitals Ethical and research Committes).

## Results

### Study population

The demographic and clinical characteristics of the study population, grouped by clinical condition, appear in Table 1. Individual´s data are enclosed as Supplementary Table S1. Mean age was significantly higher in the nonallergic control group compared to the overall allergic group and asthmatic allergic subjects. There were no differences in sex between the nonallergic and overall olive-pollen-allergic group, though the percentage of asthmatic women among the olive-pollen-allergic subjects was higher than in the group of nonasthmatic olive-pollen-allergic subjects. All groups contained both smokers and nonsmokers, although most were nonsmokers. All allergic patients had rhinitis, and 68% also had asthma. Allergic patients were graded according to disease severity as mild (33.3%) or moderate (66.7%) allergy.

**Table 1:** Demographic and clinical characteristics of the study population.

	n	Age (years)	Sex (%)	Smoking	Clinical diagnosis		Positive skin prick test	Total IgE levels (kU/l)	Specific IgE levels (kUA/l)
**Nonallergic control**	10	45.8 ± 14.7	50% female	12.5% smoking	Non-allergic		100% Negative	175.0 ± 224.8	Olive pollen IgE: 0.1 ± 0.1
			50% male	87.5% non-smoking					Ole e 1 IgE: NA
**Total Olive pollen-allergic subjects**	19	32.2 ± 12.3*	64.7% female	16.7% smoking	32% Rhinitis and no asthma	33.3% mild allergy	100% *Olea europaea* 47.4% *Phleum pratense*, 26.3% *Salsola kali*, 21.1% *Cupressus sempervirens*, 21.1% *Platanus acerifolia*, 15.8% Pets (cat/dog), 15.8% *Alternaria alternata*, 10,53% Hymenoptera, 5.26% Food allergy: egg, 5.26% *Parietaria judaica*, 5.26% LTP	425.4 ± 599.3	Olive pollen IgE: 18.2 ± 27.6
			35.3% male	83.3% non-smoking	68% Asthma and rhinitis	66.7% moderate allergy			Ole e 1 IgE: 6.9 ± 9.9
**• Nonasthmatic Olive- pollen-allergic subjects**	6	39.2 ± 13.0	33.3% female	50% smoking	100% Rhinitis	83.3% mild allergy	100% *Olea europaea* 50% *Phleum pratense*, 33.3% Hymenoptera, 16.6% LTP	282.1 ± 301.2	Olive pollen IgE: 18.4 ± 30.6
			66.7% male	50% non-smoking		16.7% moderate allergy			Ole e 1 IgE: 1.8 ± 3.6
**• Asthmatic Olive- pollen-allergic subjects**	13	28.7 ± 10.8*	81.8% female	0% smoking	100% Asthma and rhinitis	8.3% mild allergy	100% *Olea europaea* 46.2% *Phleum pratense*, 38.5% *Salsola kali*, 30.8% *Cupressus sempervirens*, 30.8% *Platanus acerifolia*, 23.1% Pets (cat/dog), 23.1% *Alternaria alternata*, 7.7% *Parietaria judaica*, 7.7% Food allergy: egg	491.5 ± 697.1*	Olive pollen IgE: 18.1 ± 22.4
			18.2% male	100% non-smoking		91.7% moderate allergy			Ole e 1 IgE: 9.2 ± 11.0

NA: not applicable. *Statistically significant differences compared to nonallergic subjects (p < 0.05).

The mean levels of total IgE were higher in the overall group of olive-pollen-allergic patients (425.4 ± 559.3 IU/ml) compared to the control group (175.0 ± 224.80 IU/ml), though this difference was not statistically significant. According to specific IgE levels, the control group was negative to olive-pollen extract specific IgE, while all allergic patients showed positive specific IgE levels (>0.35 kUA/l), with a mean of 18.22 ± 27.57 kUA/l. Furthermore, referring to recombinant Ole e 1 specific IgE, all allergic patients had positive levels (mean levels of 6.89 ± 9.89 kUA/l). There were lower Ole e 1-specific IgE levels in nonasthmatic (1.81 ± 3.54 kUA/l) than in asthmatic (9.23 ± 11.02 kUA/l) patients, but without reaching the statistical significance.

### Peptides 2 and 3 induce the lowest proliferative response in the PBMCs of olive-pollen-allergic patients

**Figure 2:**
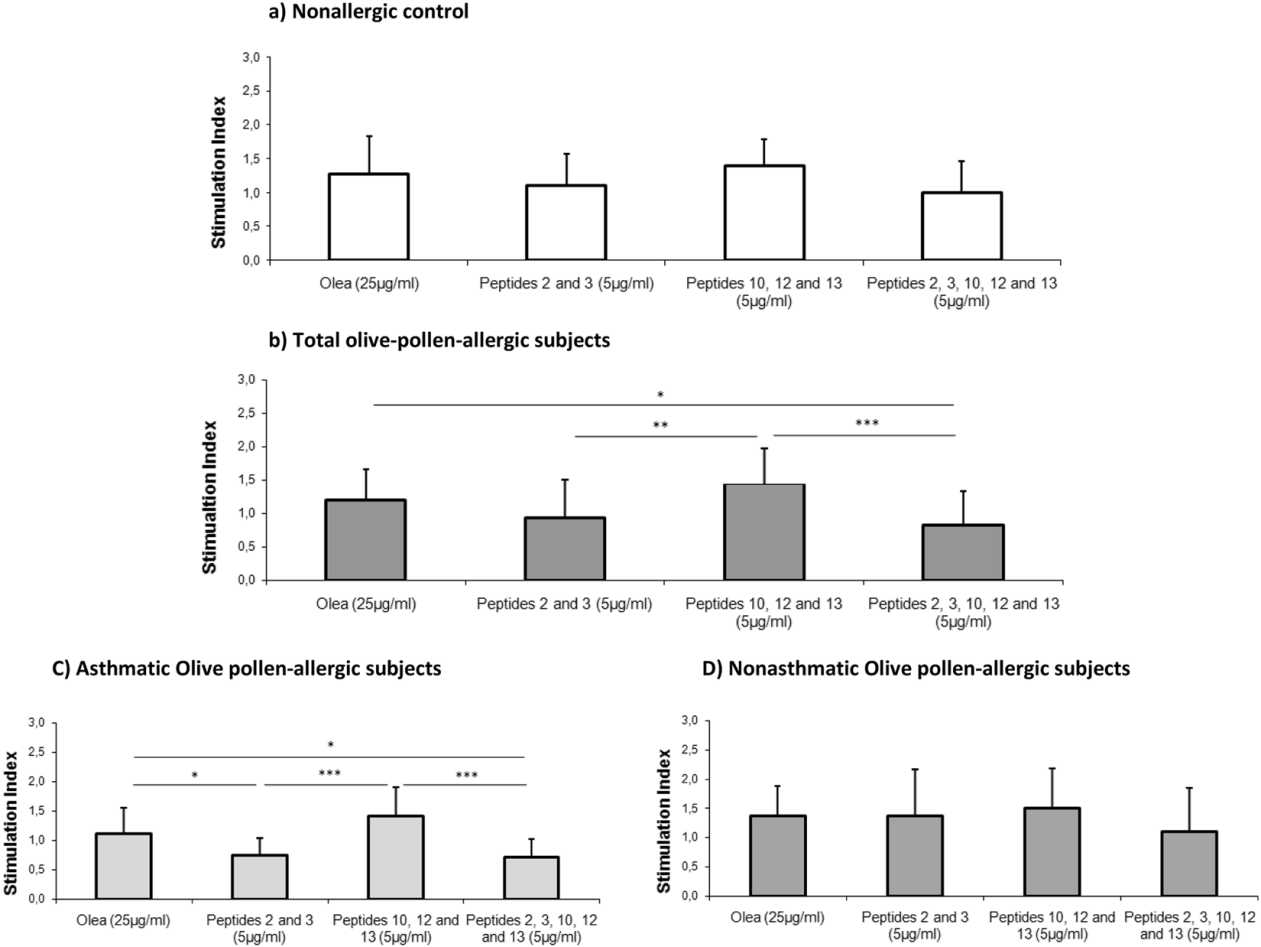
Proliferation of PBMCs from four experimental groups. (**a**) Nonallergic control (n = 9); (**b**) total olive-pollen-allergic subjects (n = 19); (**c**) asthmatic olive-pollen-allergic subjects (n = 13); and (**d**) nonasthmatic olive-pollen-allergic subjects (n = 6). The mean of stimulation index is represented for each experimental condition. Error bars represent the standard desviation of data. This stimulation index is calculated as the ratio of average absorbance of each condition and basal condition. *Statistically significant differences (p < 0.05). **Statistically significant comparison (p < 0.01). ***Statistically significant comparison (p < 0.001).

After 6 days of stimulation with olive-pollen extract or peptides, PBMC proliferation was analyzed. Figure 2a presents the results obtained in the nonallergic control group, where stimulation with peptides and olive-pollen extract did not produced any significant difference. In Fig. 2b, the results from all olive-pollen-allergic patients are represented. In this case, PBMCs stimulated with peptides 10, 12, and 13 had the highest proliferation index, producing statistically significant differences compared with P2, 3 (p = 0.0056) and P2, 3, 10, 12, and 13 (p = 0.0005). Olea stimulation also had higher proliferation index with significant differences compared to stimulation with peptides 2, 3, 10, 12, and 13 (p = 0.0142).

Figures 2c,d show the results of asthmatic olive-pollen-allergic subjects and nonasthmatic olive-pollen-allergic subjects analyzed separately. The overall behavior was the same in both groups, though in the nonasthmatic olive-pollen-allergic subjects there were no significant differences. In contrast, in the asthmatic olive-pollen-allergic group, there were significant differences between response to Olea and to peptides 2 and 3 (p = 0.0191) and also between Olea response and response to peptides 2, 3, 10, 12, and 13 (p = 0.0140). There were statistically significant differences between stimulation with peptides 10, 12, 13 and stimulation with peptides 2 and 3 (p = 0.0006) and the combination of all peptides (p = 0.0002).

### Prestimulation with peptides 2 and 3 before olive-pollen-extract stimulation reduces PBMC proliferation in olive-pollen-allergic patients

**Figure 3:**
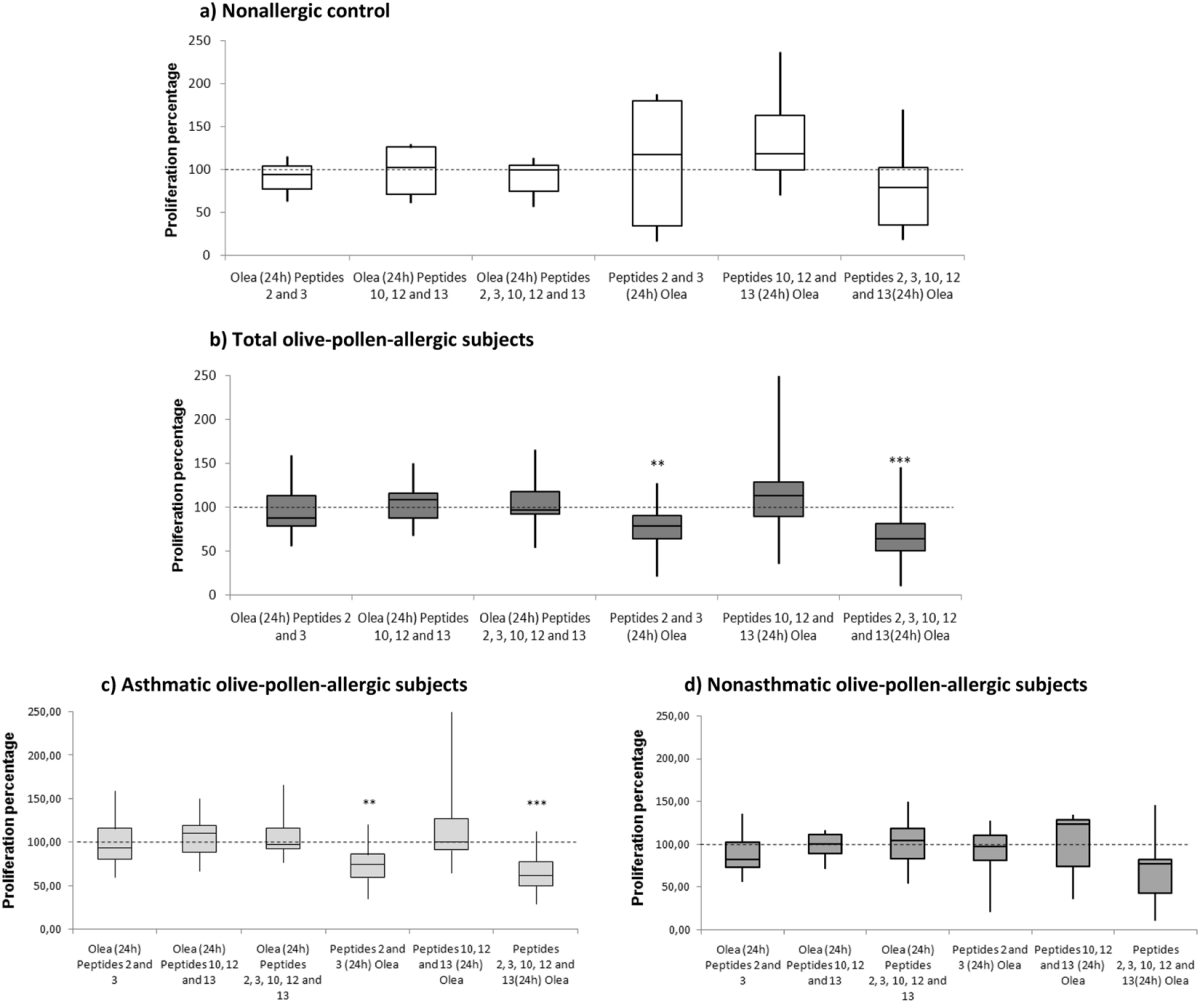
Inhibition results of PBMCs response to olive pollen extract with immunomodulatory peptides. 100% proliferation was assigned to olive pollen extract (Olea) (dotted line). Results are shown in a box and whisker plot, with the mean, standard deviation, maximum and minimum values for the different groups: (**a**) nonallergic control (n = 9), (**b**) total olive-pollen-allergic subjects (asthmatic and nonasthmatic) (n = 19), (**c)** asthmatic olive-pollen-allergic subjects (n = 13); and (**d**) nonasthmatic olive-pollen-allergic subjects (n = 6). **Statistically significant comparison between each combination and Olea condition (p < 0.01). ***Statistically significant differences (p < 0.001).

Once we observed the low proliferative response induced by peptides 2 and 3 to PBMCs of allergic subjects, we then studied the capacity of these peptides to avoid or reverse PBMC proliferation in response to olive-pollen-extract stimulation. To do this, PBMCs were exposed to peptides 2 and 3, peptides 10, 12, and 13, or both groups of peptides either 24 hours before (inhibition or prevention of allergic response) or after (reversion of allergic response) olive-pollen-extract stimulation (Fig. 1b,c). The proliferation obtained in these conditions was compared with PBMCs stimulated only with olive-pollen extract. Figure 3 contains the results of these experiments.

Figure 3a depicts the results obtained in the nonallergic control group, where no differences were found between peptides and olive-pollen extract. However, olive-pollen-allergic patients showed a different pattern (Fig. 3b). Although stimulation with peptides after exposure to the whole extract caused no significant effect in proliferation—that is, producing an effect similar to the one obtained with olive-pollen extract only—differences were observed when peptides were administered before exposure to the extract; specifically, peptides 2 and 3 and peptides 2, 3, 10, 12, and 13 prevented the olive-pollen-derived proliferation of PBMCs. In both cases there was a significant decrease in proliferation after olive-pollen-extract stimulation; specifically, peptides 2 and 3 induced up to 22.8% inhibition (p = 0.0029), and the combination of all the peptides together—i.e. peptides 2, 3, 10, 12, and 13—caused up to 32.7% inhibition (p = 0.0003) when the peptides were administered first. These data could indicate that the peptides are more capable of preventing the olive-pollen-allergen response than inhibiting a previously established response.

As stated above, separate study of the asthmatic and nonasthmatic patients showed differences in peptide response in both groups (Fig. 3c,d). Peptides administered after olive-pollen stimulation could not revert the proliferative response in either group. However, the different combinations of peptides (peptides 2 and 3 or peptides 2, 3, 10, 12, and 13), when applied before the extract, reduced the proliferative response in both groups, though statistically significant differences were only found in asthmatic patients. Peptides 2 and 3 and peptides 2, 3, 10, 12, and 13 induced a statistically significant decrease in the response to Olea, with 28.27% inhibition (p = 0.0011) and 34.79% inhibition (p = 0.0003), respectively. In nonasthmatic patients, the reduction induced by the five peptides did not reached the statistical significance.

### Stimulation with Ole e 1-derived peptides (peptides 2 and 3) induces the secretion of IL-10 and IL-35

IL-10 secretion was measured in the nonallergic control group and overall olive-pollen-allergic patients. In both groups, there were several samples that showed undetectable levels of IL10, especially in the control group. The mean IL-10 levels were slightly higher in basal samples from allergic patients (36.265 ± 31.7 pg/ml) compared to controls (12.776 ± 12.51 pg/ml), but without statistically significant differences.

According stimuli, the behavior was similar in both groups, as peptides 2 and 3 alone or in combination with peptides 10, 12, and 13 showed the highest levels of IL-10. However, healthy controls did not show significant differences between any conditions.

**Figure 4:**
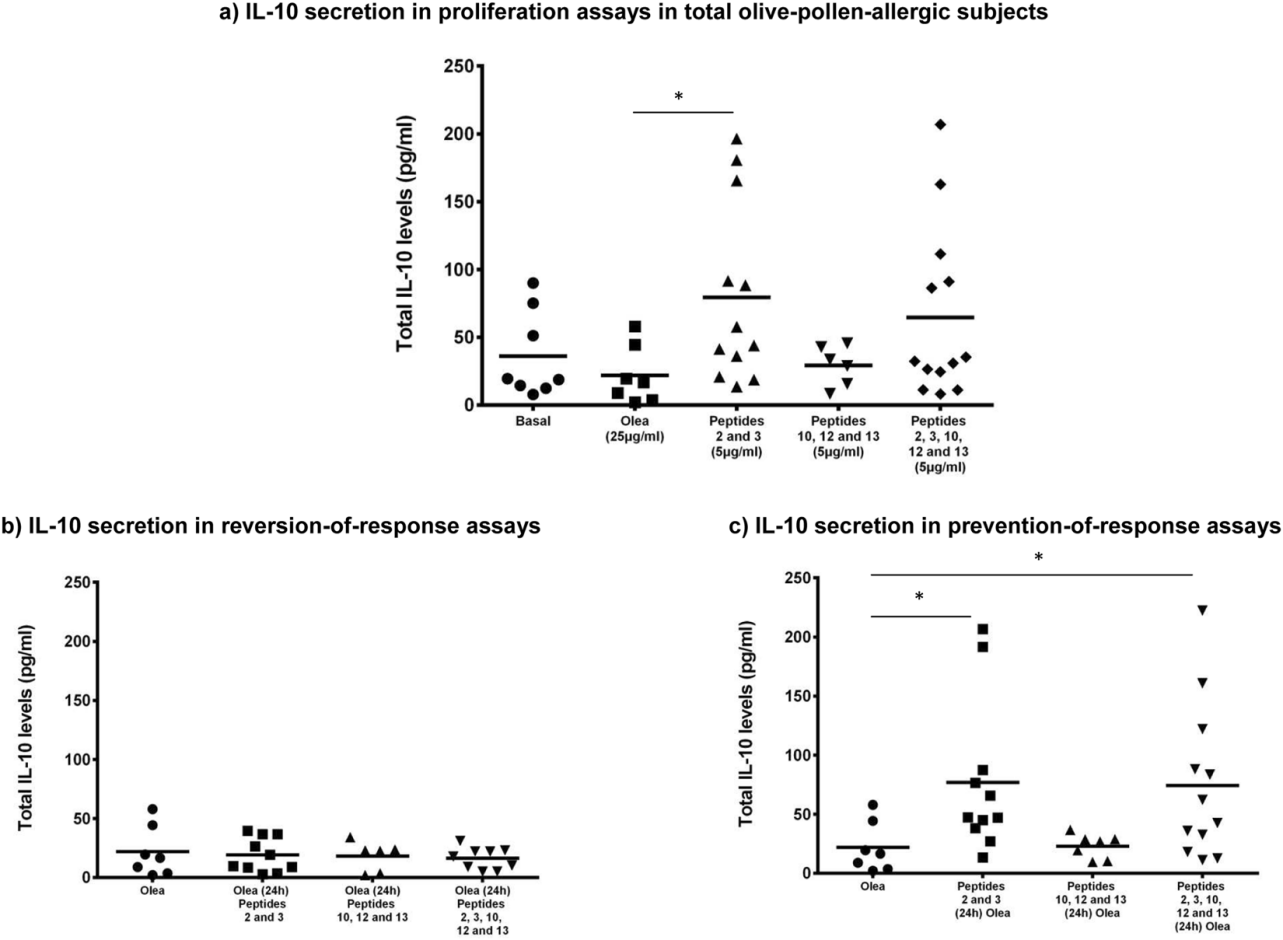
IL-10 regulatory cytokine levels in supernants of PBMCs from olive-pollen-allergic patients. *Statistically significant comparison p < 0.05.

The most remarkable results were seen in the olive-pollen-allergic patients. In this group, following stimulation with peptides 2 and 3, IL-10 secretion increased significantly (79.64 ± 66.13 pg/ml) compared to stimulation in response to olive-pollen exposure (21.94 ± 21.35 pg/ml), p = 0.0283. The combination of peptides 2 and 3 with peptides 10, 12, and 13 (64.62 ± 63.36 pg/ml) also produced an increase in IL-10 induction, but without reaching statistical significance (Fig. 4a). The reversion-of-response assays showed no differences in IL-10 secretion (Fig. 4b). However, pre-stimulation (Fig. 4c) with peptides 2 and 3 (76.97 ± 64.04 pg/ml) alone and in combination (74.41 ± 65.53 pg/ml) 24 hours before olive-pollen stimulation, induced significantly higher secretion of IL-10, compared to Olea (21.94 ± 21.35 pg/ml) (p = 0.014 and 0.036, respectively).

For control subjects none condition showed statistical significant differences. Stimulation with peptides 2 and 3 (63.54 ± 62.69 pg/ml) and with the five peptides (55.5 ± 73.4 pg/ml) increased the IL-10 secretion compared with olive-pollen stimulation (7.03 ± 0.001 pg/ml) but without statistical significant differences. The reversion assays showed similar data than stimulation with Olea alone (7.03 ± 0.001 pg/ml) after addition of peptides 2 and 3 (8.23 ± 8.39 pg/ml) and after stimulation with all the peptides (7.04 ± 6.65 pg/ml). Finally, the reversion assays results in control subjects ranged from 30.5 ± 47.7 pg/ml with peptides 2 and 3 pre-stimulation to 43.21 ± 58.3 pg/ml after pre-stimulation with all the peptides, but without any statistical significant result.

As IL-10 and IL-35 are closely related, and given that there is a relationship between IL-35 and Treg cells, we also studied the effect of the selected peptides on the secretion of this interleukin. All the samples from control subjects and olive-pollen allergic patients showed detectable levels of IL35. The mean IL-35 levels were lower in basal samples from allergic patients (119.47 ± 259 pg/ml) compared to controls (287.55 ± 483 pg/ml), but without statistically significant differences. According to the stimuli, the behavior was similar in both groups, but with much higher levels in allergic patients than in controls, being these differences statistically significant in olive-pollen allergic patients stimulated with Olea samples (333.62 ± 483 pg/ml) compared to control subjects (49.28 ± 50.93 pg/ml), and in reversion with peptides 2, 3 (537.9 ± 368 pg/ml in olive-pollen allergic patients *vs*. 217.8 ± 357 pg/ml, in control group) and with peptides 2, 3, 10, 12, 13 (618.3 ± 440 pg/ml in olive-pollen allergic patients *vs*. 262.72 ± 380 pg/ml, in control group).

**Figure 5:**
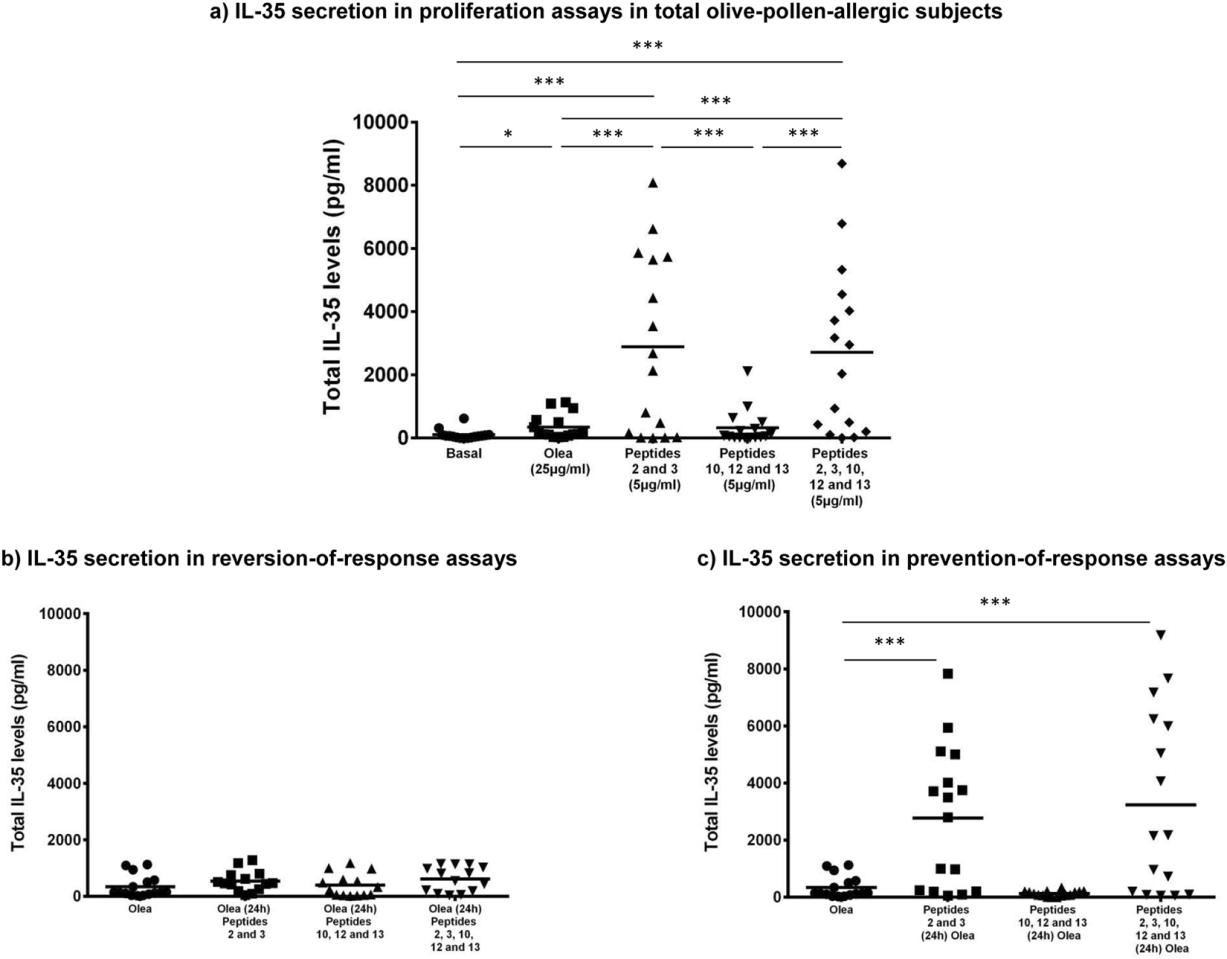
IL-35 regulatory cytokine levels in supernants of PBMCs from olive-pollen-allergic patients. *Statistically significant comparison p < 0.05. ***Statistically significant comparison, p < 0.001.

As in the IL-10 analysis, the results shown correspond to the IL-35 secreted in proliferation (Fig. 5a), reversion (Fig. 5b) and prevention-of-response assays (Fig. 5c) in the olive-pollen-allergic patients. Again, stimulation with peptides 2 and 3 (2717.57 ± 2807.12 pg/ml) and peptides 2, 3, 10, 12, and 13 (2555.03 ± 2617.68 pg/ml) showed a significant increase in IL-35 secretion, compared to basal (119.79 ± 259.32 pg/ml), Olea (331.97 ± 413.88 pg/ml) and peptides 10, 12, and 13 (301.02 ± 526.64 pg/ml) being all these difference extremely significance (p < 0.0001). In control group stimulation with peptides 2 and 3 (1369.03 ± 1918.19 pg/ml) and peptides 2, 3, 10, 12, and 13 (1229.11 ± 1541.31 pg/ml) showed statistical significant increase (p < 0.05) in IL-35 secretion, compared to basal (287.55 ± 483 pg/ml), Olea (49.28 ± 50.93 pg/ml) and peptides 10, 12, and 13 (126.74 ± 279.74 pg/ml). Additionally, the addition of peptides 24 hours latter than the olive pollen extract was unable to induce a statistically significant increase in the IL-35 secretion. However, the olive-pollen response prevention assays induced an increase in IL-35 levels (Fig. 5c). Pre-stimulation with peptides 2 and 3 (2776.84 ± 2456.34 pg/ml) increased the secretion of this interleukin by up to 8 times compared to olive pollen (p < 0.0001). In the case of the combination of peptides 2 and 3 with 10, 12, and 13 (3241.41 ± 3203.66 pg/ml), this increase was almost 10-fold (p < 0.0001). In control group, pre-stimulation with peptides 2 and 3 (1546.18 ± 1948.11 pg/ml) and the combination of peptides 2 and 3 with 10, 12, and 13 (1337.62 ± 1863.37 pg/ml) also an statistical significant increase (p < 0.05) of the IL-35 levels was showed compared to olive pollen stimuli.

### Peptides are not capable of inducing basophil activation *in vitro*

**Figure 6:**
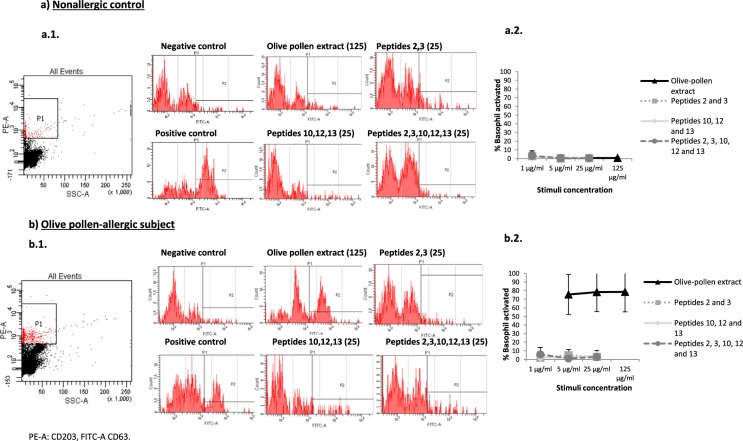
Basophil activation test. (**a**) Nonallergic control subjects (n = 3). (**b**) Olive-pollen-allergic patients (n = 3). a1 and b1 are representative examples of histograms obtained with the different stimuli. a2 and b2 show the mean levels of the percentage of basophils activated in response to three different concentrations of each stimulus used. Activation threshold (>15%).

Basophil activation was analyzed to test the safety of the peptides *in vitro*. We collected blood samples from 3 nonallergic subjects and 3 olive-pollen-allergic patients. Then, we tested 3 different concentrations of olive-pollen extract, that is, peptides 2 and 3 and/or peptides 10, 12, and 13 (see Fig. 6 for results). In all cases, positive controls showed higher than 50% activation. In the nonallergic control samples, neither stimulus produced basophil activation higher than 15% (Fig. 6a). Furthermore, olive-pollen extract produced more than 50% basophil degranulation in all the olive-pollen-allergic samples, while the different combinations of peptides did not produce basophil activation (Fig. 6b).

## Discussion

This report tries to demonstrate the immunoregulatory ability of peptides derived from the major allergen of olive pollen, in order to define new therapeutic tools for improving therapy for olive-pollen allergy. The sequence of the peptides used was derived from the “canonical” sequence of Ole e 1 obtained after Edman degradation^18^. These peptides were previously defined as immunodominant (peptides 10, 12, and 13) and as possible immunoregulatory T cell-epitopes referring to peptides 2 and 3 (aa11-33) and were characterized as stimuli that directly influence gene-expression profile, in particular modulating immunoregulatory genes^17,19,20^. Ole e 1 has been described as a polymorphic glycoprotein^13,14^ where a total of 21 positions out of 145 amino acids of Ole e 1 are replaced, approximately 15% of the whole sequence, depending of the olive cultivar studied^14^. Four of these amino acid substitutions (at positions 91, 95, 99, and 110) were related with our immunodominant peptides (included in peptides 10 and 12). For that, one of the possible limitations of this report could be due to the design of the peptides. However, those aa changes mainly could be related with the tertiary structure, and to be important for B cell epitopes but in the case of T cell epitopes (short peptides) this aspect could be relevant mainly if the amino acidic changes were related with the anchor peptides for HLA class II binding. This aspect was previously checked by a T cell epitopes free program (www.imtech.res.in/raghava/propred/) and the aa changes described were not essential for HLA class II restriction. However, the relevance of the Ole e 1 isoforms is a very relevant aspect of the complexity in the olive pollen response, mainly related with the high number of olive species cultivated. This high diversity has been associated with Ole e 1 polymorphic sequences and this fact could have important clinical repercussion, at diagnostic as well as treatment levels, as has been previously discussed^23^. This matter should be studied in more detail and in other allergens^24^ for a better management of this complex respiratory disease.

The use of short-length peptides derived from T-cell epitopes is being studied extensively as a new approach in the field of immunotherapy. This kind of peptides was recently named SPIRE because of their capacity to induce tolerance. The main mechanisms implicated in this approach are based on the lack of conformational epitopes. They are designed to induce immunological tolerance by binding to MHC class-II molecules on antigen-presenting cells. These peptides induce Treg cells, possibly triggering the production of IL-10, TGF-β and inducing Foxp3 expression and increased Th1 response^6,8,25^.

One important consideration for therapy safety is that SPIRE should not bind to IgE-FcεRI on effector cells, so as to prevent them from cross-linking to IgE. This provides an advantage by potentially reducing the risk of IgE-mediated allergic reactions such as rhinitis, asthma, and pruritus^11^. For this reason, all candidate peptides must be tested both alone and in combination to ensure their inability to bind and cross-link inflammatory cell-bound IgE. A convenient and reliable assay to assess the clinical and functional relevance of IgE reactivity is the basophil activation test^26^. Using this assay, we demonstrated that the Ole e 1-derived peptides used in this study did not bind to IgE (Fig. 6).

The study was carried out in a total of 29 subjects. These individuals were recruited and characterized according to their clinical symptoms, the results of skin prick testing for conventional allergens and humoral responses. These tests revealed that the highest levels of total and specific IgE were found in asthmatic patients (Table 1). PBMCs were challenged *in vitro* with different synthetic Ole e 1 dodecapeptide combinations as well as the whole allergenic olive-pollen extract. Proliferation assays are used to identify T-cell epitopes and to test for specific response to allergens, and also to develop new approaches in immunotherapy such as SPIRE^27–30^.

Proliferation assays showed that peptides 2 and 3 induced the lowest proliferative response in the olive-pollen-allergic PBMCs compared to the whole olive-pollen extract and immunodominant peptides 10, 12, and 13 (Fig. 2). A further step involved to design a mechanism of proliferation inhibition to evaluate the capacity of these peptides to revert or prevent the response to the olive-pollen extract. Figure 3b shows how neither peptide with this design could reverse a previously established response by olive-pollen extract. However, peptides 2 and 3 partially prevented the total cellular response to olive-pollen extract, reducing this response to 22.8% when assayed alone and 32.7% when used in combination with peptides 10, 12, and 13, as shown by the significant decrease observed in the stimulation index of PBMCs from the untreated allergic subjects. Our analysis of PBMC response in allergic patients by clinical phenotype (asthmatic *vs* nonasthmatic) showed that the inhibition of peptides 2 and 3 was greater in the asthmatic patients than in the nonasthmatic subjects (Fig. 3c,d). All these data are in agreement with our previous results demonstrating how peptides 2 and 3 modified the gene expression profile of untreated olive-pollen-allergic patients, mainly in those genes that are essential to maintaining the peripheral T-cell tolerance and which play a key role in the inflammatory response^19^.

Closely related with the regulatory response, some cytokines have a particular function controlling the immune system response. This study analyzed the implication of classical and novel regulatory cytokines, such as IL-10 and IL-35, in this modulation. IL-10 is secreted by Treg cells, which inhibits the expansion of Th2 response and blocks the production of IgE and mucus and the migration of effector cells to the tissues affected by allergy^30^. Peptides 2 and 3—alone and in combination with immunodominant peptides—induced IL-10, increasing the microenvironment that benefits immunotolerance in untreated allergic subjects (Fig. 4a). Additionally, as happened with the proliferation assays, these peptides were unable to induce the regulatory cytokines when were added after olive pollen extract (Fig. 4b). However, the stimulation with these peptides before exposure to the olive-pollen extract maintains the high levels of this cytokine (Fig. 4c). These results are in accordance with the high levels of IL-35 found after stimulation with peptides 2 and 3 (Fig. 5a,c), except in the reversion assays (Fig. 5b). These data remark that Ole e 1 peptides could induce a regulatory response, mediated by IL-35 and IL-10, able to reduce the response against to olive pollen, indicating that these peptides could be useful as preventive therapeutic tools for these responses.

IL-35 is secreted mainly by stimulated Tregs^21^. It is a heterodimer composed of IL-12p35 and EBI3 subunits^22^, this last, one of the genes that we found as specifically modulated by olive-pollen peptides 2 and 3 and considered as a possible therapeutic target for olive-pollen allergy^20^. The functional implications of this cytokine are now being extensively studied to describe its immunosuppressive activity in different inflammatory and autoinmune diseases, mainly in animal model^31–33^. IL-35 promotes the development of regulatory T cells (Tregs) and regulatory B cells (Bregs) and very recently, has been described the correlation between IL-10 and IL-35 by the induction of phosphorylation of STAT1 and STAT3 in B cells, promoted by IL-35^34^. However, few studies of IL-35 in relation with allergic diseases have been performed. Recent findings suggested that expression of IL-35 is abnormal in asthma, playing an important role in the pathogenesis of this disease^35^. It has also been described how people with asthma and chronic obstructive pulmonary disease (COPD) have low levels of this cytokine, though serum levels of the cytokine increased after immunotherapy, marking an effect associated with an improvement of clinical symptoms^36^. Finally, according with our results, very recently^37^ IL-35 and inducible Tregulatory 35 cells have been described as induced by sublingual allergy immunotherapy, pointing that IL-35 therapy could be useful for treatment of respiratory allergic diseases^38^. For that, despite the limited number of patients included, the results of this study are very promising.

## Conclusion

We have demonstrated that the combination of five short dodecapeptides Ole e 1 derived-peptides is able to prevent the olive-pollen proliferative response associated to IL-10 and IL-35 regulatory cytokines production in allergic patients. Moreover, these combinations of peptides are not capable of inducing basophils activation, a pre-requisite for the development of a new peptide vaccine. Further basic and clinical studies are needed to broaden knowledge of these capacities and to confirm their possible use in routine clinical practice. Now, however, peptides hold clear potential as new tools for olive-pollen-allergy specific immunotherapy.

## Supplementary information

Sypplementary Fig. 1 and Table 1
