# Supplementary material for: Therapeutic potential of peptides from Ole e 1 in olive-pollen allergy
Source: Sci Rep. 2019 Nov 4;9:15942. doi: 10.1038/s41598-019-52286-3 (PMC6828773; doi:10.1038/s41598-019-52286-3)
Supplement: Supplementary file 1 — Sypplementary Fig. 1 and Table 1 [file 41598_2019_52286_MOESM1_ESM.pdf]

Supplementary material associated with:

Title: Therapeutic potential of peptides from Ole e 1 in olive-pollen allergy

Authors: ***David Calzada[1], *Lucía Cremades-Jimeno[1], María Ángeles de Pedro[1], Selene Baos[1], Manuel Rial[2,3], Joaquín Sastre[2,3], Joaquín Quiralte[4], Fernando Florido[5], Carlos Lahoz[1, 3], Blanca Cárdaba[1, 3].**

[*]D. Calzada and L. Cremades-Jimeno have contributed equally to this article.

Institutions:
[1]Immunology Department, IIS-*Fundación Jiménez Díaz*, UAM, Madrid, Spain.
[2]Allergy Department, *Fundación Jiménez Díaz*, Madrid, Spain.
[3]CIBERES, CIBER of Respiratory Diseases, Spain.
[4]Allergy Department, *Vírgen del Rocío* University Hospital, Seville, Spain.
[5]Allergy Department, *San Cecilio* University Hospital, Granada, Spain.

Correspondence: Blanca Cárdaba
Immunology Department
IIS-*Fundación Jiménez Díaz*
Avda Reyes Católicos Nº 2
28040 Madrid
Spain

Telephone/FAX: +34-91-5494445/ +34-91-5448246
E-mail address: bcardaba@fjd.es

Supplementary Figure S1. Time-curve to determine optimal inhibition conditions of PBMCs response to olive pollen extract with immunomodulatory peptides. PBMCs from nonallergic control (n=3) and olive-pollen-allergic subjects (n=3) were cultured with different combinations of peptides or/and olive-pollen extract (Olea) as is indicated in the figure, for 6 days, in order to determine: the reversion of the response or the inhibition response (after 24 or 48 hrs of pre-stimulation). Three different combinations of peptides were used: peptides 2 and 3 (immunoregulatory region); peptides 10, 12, and 13 (immunodominant region), and both groups of peptides. After 6 days of stimulation, proliferative response was measured. 100% proliferation was assigned to olive pollen extract (Olea) (dotted line). Results are shown in a box and whisker plot, with the mean, standard deviation, maximum and minimum values for the different groups: Upper graph, nonallergic control (n=3) and lower graph, olive-pollen-allergic subjects (n=3). *Statistically significant comparison between the indicated combination and Olea condition ($p<0.05$). **Statistically significant comparison between the indicated combination and Olea condition ($p<0.01$).

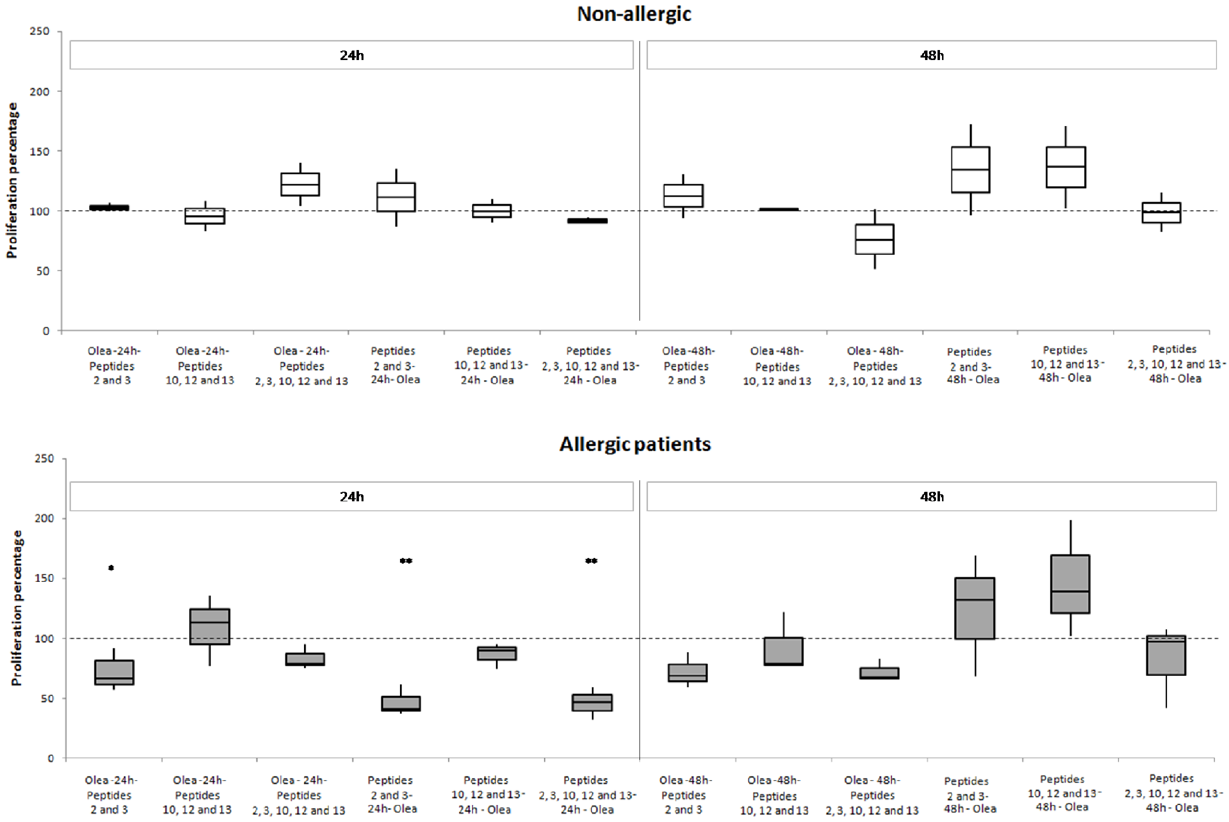

Supplementary Table S1. Demographic and clinical characteristics of each individual studied.

Patient number	Age (years)	Sex	Smoking	Clinical diagnosis			Skin prick test	Total IgE levels (kU/l)	Specific IgE levels (Olive pollen) (kUA/l)	Specific IgE levels (Ole e 1) (kUA/l)
				Allergy severity	Rhinitis	Asthma				
HC-1	49	Female	Yes	Control	No	No	Negative	374.00	0.00	NA
HC-2	52	Male	No	Control	No	No	Negative	147.00	0.00	NA
HC-3	66	Male	Former	Control	No	No	Negative	24.50	0.00	NA
HC-4	41	Female	No	Control	No	No	Negative	ND	ND	NA
HC-5	60	Female	No	Control	No	No	Negative	44.30	0.00	NA
HC-6	55	Female	No	Control	No	No	Negative	14.50	0.00	NA
HC-7	21	Male	No	Control	No	No	Negative	31.50	0.02	NA
HC-8	45	Male		Control	No	No	Negative	168.00	0.34	NA
HC-9	22	Female	No	Control	No	No	Negative	77.90	0.00	NA
HC-10	47	Male		Control	No	No	Negative	693.00	0.07	NA
A-1	26	Male	Yes	Mild	Yes	No	*Olea europaea*, Hymenoptera, LTP allergy	168.00	0.68	0.01
A-2	29	Male	No	Mild	Yes	No	*Olea europaea*	789.00	96.70	0.00
A-3	52	Female	No	Moderate	Yes	No	*Olea europaea*, Hymenoptera allergy	76.40	1.37	0.56
A-4	49	Male	Yes	Mild	Yes	No	*Phleum pratense*, *Olea europaea*	46.20	0.04	0.02
A-5	27	Male	Yes	Mild	Yes	No	*Phleum pratense*, *Olea europaea*	101.00	1.41	0.56
A-6	52	Female	No	Mild	Yes	No	*Phleum pratense*, *Olea europaea*	512.00	10.40	9.71
A-7					Yes	Yes	*Olea europaea*	159.00	33.50	13.20
A-8	28	Female	No	Moderate	Yes	Yes	*Olea europaea*	407.00	40.00	26.90
A-9	32	Female	No	Moderate	Yes	Yes	*Olea europaea*	37.00	1.54	1.17
A-10	50	Female	No	Moderate	Yes	Yes	*Olea europaea*	38.80	7.51	4.74
A-11	42	Female	Former	Moderate	Yes	Yes	*Olea europaea*	159.00	6.22	6.88
A-12	30	Female	No	Moderate	Yes	Yes	*Olea europaea*, *Salsola kali*	41.60	>100	0.08
A-13	21	Male	No	Moderate	Yes	Yes	*Cupressus sempervirens*, *Olea europaea*, *Parietaria judaica*, *Phleum pratense*, *Salsola kali*; urticaria to almond	14.30	0.73	0.67
A-14	23	Female	No	Mild	Yes	Yes	*Cupressus sempervirens*, *Olea europaea*, *Phleum pratense*, *Platanus acerifolia*	112.00	2.24	1.78
A-15	16		No	Moderate	Yes	Yes	*Alternaria alternata*, *Olea europaea*, *Platanus acerifolia*, *Salsola kali*, animals (cat, dog, horse)	1676.00	47.10	23.30
A-16	18	Female	No	Moderate	Yes	Yes	*Cupressus sempervirens*, *Olea europaea*, *Phleum pratense*, *Platanus acerifolia*, *Salsola kali*	1066.00	65.40	31.20
A-17	29	Male	No	Moderate	Yes	Yes	*Alternaria alternata*, *Olea europaea*, *Phleum pratense*	2149.00	1.19	0.03
A-18	16	Female	No	Moderate	Yes	Yes	*Cupressus sempervirens*, *Olea europaea*, *Phleum pratense*, *Platanus acerifolia*, *Salsola kali*, animals (cat, dog, horse), food allergy (egg); atopic dermatitis, urticaria to shrimp	397.00	10.50	8.67
A-19	39	Female	No	Moderate	Yes	Yes	*Alternaria alternata*, *Olea europaea*, *Phleum pratense*, animals (cat, dog); AINES intolerance	133.00	1.37	1.42

HC: Healthy Controls Subjects. A: Olive-pollen-allergic patients. NA: not applicable. ND: not determined.
